# Cortical processing and perceived timing

**DOI:** 10.1098/rspb.2007.0576

**Published:** 2007-07-10

**Authors:** Derek H Arnold, Paul Wilcock

**Affiliations:** School of Psychology, The University of QueenslandSt Lucia, Queensland 4072, Australia

**Keywords:** timing perception, cortical processing, consciousness

## Abstract

As of yet, it is unclear how we determine relative perceived timing. One controversial suggestion is that timing perception might be related to when analyses are *completed* in the cortex of the brain. An alternate proposal suggests that perceived timing is instead related to the point in time at which cortical analyses *commence*. Accordingly, timing illusions should not occur owing to cortical analyses, but they could occur if there were differential delays between signals reaching cortex. Resolution of this controversy therefore requires that the contributions of cortical processing be isolated from the influence of subcortical activity. Here, we have done this by using binocular disparity changes, which are known to be detected via analyses that originate in cortex. We find that observers require longer stimulus exposures to detect small, relative to larger, disparity changes; observers are slower to react to smaller disparity changes and observers misperceive smaller disparity changes as being perceptually delayed. Interestingly, disparity magnitude influenced perceived timing more dramatically than it did stimulus change detection. Our data therefore suggest that perceived timing is both influenced by cortical processing and is shaped by sensory analyses subsequent to those that are minimally necessary for stimulus change perception.

## 1. Introduction

A single physical event can induce activity that is widely distributed both in time and across different, specialized, sensory regions of cortex (Zeki 1978; Bullier 2001). The temporal distribution of neural activity related to a common physical event raises the possibility that we might exist in patchwork perceptual moments pieced together from different epochs at whatever rate the brain can sustain (Moutoussis & Zeki 1997; Bartels & Zeki 1998; Whitney & Murakami 1998; Arnold *et al*. 2001).

While it is generally accepted that timing illusions can be instigated by differential delays between physical exposure to a stimulus and the onset of activity in cortex (Roufs 1963; Wilson & Anstis 1969), the possibility that subsequent processing *within* cortex might also contribute is more controversial (Dennet & Kinsbourne 1992; Johnston & Nishida 2001; Nishida & Johnston 2002). Many sensory judgements require that cortical activity be first integrated over time before a sensory threshold is achieved (Bartels & Zeki 1998; Van de Grind 2002). This necessity could influence timing judgments. However, such a relationship between cortical processing and perceived timing could cause unnecessary timing errors (Dennet & Kinsbourne 1992; Johnston & Nishida 2001; Nishida & Johnston 2002). Hypothetically, these errors could be eliminated if perceived timing were related to when cortical analyses *commence* rather to when they *finish* (Libet *et al*. 1979; Eagleman & Sejnowski 2000; Rao *et al*. 2001; Moradi & Shimojo 2004; Amano *et al*. 2007).

Determining whether there is a relationship between cortical processing and perceived timing can be problematic as it can be difficult to tease apart the contributions of cortical activity from that which occurs in subcortical structures. It is, however, possible to isolate the influence of cortical processing by examining a stimulus change that can only be detected via analyses that originate in cortex and which is not confounded with other cues that are detectable by subcortical mechanisms.

Analyses of several visual attributes originate in cortex—orientation, spatial frequency and binocular disparity provide a few examples (Hubel & Wiesel 1962, 1968; Barlow *et al*. 1967; Poggio & Fischer 1977). However, cortical mechanisms receive input via subcortical structures where neurons can provide a transient response to local changes in luminance and colour (Derrington & Lennie 1984; Derrington *et al*. 1984; Lee *et al*. 1989; Merigan *et al*. 1991). Most simple visual events are coupled with such changes so that the timing of these events could be signalled by either cortical or subcortical mechanisms. However, it is possible to break this confound (and thereby isolate any cortical contribution to timing perception) by using a specific form of binocular disparity.

Binocular disparity occurs because our eyes are separated and receive slightly different retinal images. For most observers, these differences provide cues concerning the three-dimensional structure of the surrounding environment. For instance, uncrossed horizontal binocular disparity (which is used in the following experiments) can create an impression of a field of dots floating in space behind the point at which the observer is fixated (Julesz 1971).

Binocular disparity can only be detected following the temporal integration of disparity signals (Uttal *et al*. 1975; Tyler 1991) which become available only in cortex (Barlow *et al*. 1967; Poggio & Fischer 1977). By using a dynamic binocular display, it is possible to generate a timing cue that can only be detected via cortical analyses and which is not confounded with monocular cues that can be encoded by subcortical activity (Julesz 1971). Any influence of disparity magnitude on timing judgements using this sort of stimulus would therefore clearly demonstrate a tight relationship between the temporal integration of cortical activity and perceived timing.

## 2. Material and methods

### (a) General methods

Four observers, two of whom were naive as to the purpose of the study and the authors, participated in each experiment. All observers had normal, or corrected to normal, visual acuity, colour and stereo vision.

Visual stimuli were generated using Matlab software to drive a ViSaGe stimulus generator (Cambridge Research Systems) and were displayed on a gamma corrected 21″ Samsung SyncMaster 1100p+ monitor (1024×768 resolution; 120 Hz refresh rate). All stimuli were viewed, from a distance of approximately 78 cm, through an individually adjusted mirror stereoscope. The stimuli were presented to both the left and right eyes and surrounded by pink (CIE 1931; *X*=0.23, *Y*=0.10, luminance=14.45) frames. Black central fixation points and crosses were presented to both the left and right eyes.

In this experiment, it is essential to use a stimulus wherein the binocular change is not coupled with any monocular cues so that we can be sure that we are manipulating a stimulus change that is detected via a cortical analysis. For this reason, we used a stimulus consisting of dynamic noise patterns (3.5°×3.5°, element size 0.035° square) updated at the monitor refresh rate (120 Hz). Disparity changes in this type of stimulus are not coupled with detectable monocular changes (Julesz 1971). Uncrossed horizontal binocular disparity (0°–0.28°) was generated by shifting the dynamic elements in the left eye stimulus to the *left* relative to these in the right eye stimulus. This generated uncrossed disparity which, when perceived, made the field of dynamic dots appear to be positioned further away from the observer relative to the central fixation points and surrounding frames. When there was no disparity signal, the field of dynamic dots appeared to be in the same depth plane as the fixation point.

### (b) Experiment 1

In experiment 1, a two-interval forced choice task was used to determine temporal thresholds for detecting transient disparity signals—an objective signal detection paradigm. Each of the sequential intervals persisted for 1 s and were separated by a 500 ms interstimulus interval (ISI). The test stimulus consisted of a 1 s interval incorporating a period (0–200 ms) during which one of four magnitudes of uncrossed horizontal binocular disparity (0.07°, 0.14°, 0.21° and 0.28°) was presented. Presentation of the disparity signal was centred within the interval. The comparison stimulus consisted of a 1 s interval during which dynamic random noise was presented without a disparity signal. Interval order presentation was randomized on a trial by trial basis. On each trial, observers were required to indicate, by pressing one of two response buttons, which of the two intervals contained the test stimulus. Feedback was provided via high or low tones to indicate response accuracy.

Data from each run of trials in experiment 1 provided distributions of correct test stimulus interval detection as a function of binocular disparity signal duration. Weibull functions were fitted to these distributions and the 75% points taken as temporal threshold estimates for binocular disparity detection. Temporal threshold differences (TTDs) were calculated relative to the observers' temporal threshold estimate for the smallest disparity signal. Negative values therefore indicate that disparity changes could be detected following *shorter* stimulus presentations relative to 0.07° disparity changes.

### (c) Experiment 2

In experiment 2, a simple reaction time (RT) task was used. Each trial consisted of 1.8 s presentations of dynamic random noise. On 80% of trials, binocular disparity signals of variable magnitude (0.07°, 0.14°, 0.21° and 0.28°) were introduced at the halfway point of the stimulus presentation. On the remaining 20% of trials, no disparity signal was presented. Observers were required to press a response button, as quickly as possible, if they detected a binocular disparity signal. If no disparity was detected by the end of the stimulus presentation, observers pressed a second response button. A conservative response criterion was enforced by informing observers that if they falsely reported disparity on more than 5% of the trials in which no disparity signal was presented, their data would be rejected and the experiment repeated. None of our observers exceeded this criterion.

Data from each run of 500 trials in experiment 2 provided RT distributions for each of the four magnitudes of horizontal disparity change (0.07°, 0.14°, 0.21° and 0.28°). RT differences were calculated relative to the observers’ RTs for the smallest disparity signal. RT differences as a function of disparity magnitude are shown in figure 1*c*,*d*.

### (d) Experiment 3

In experiment 3, a forced choice temporal synchrony judgement was used—a subjective measure of timing. At the start of each trial, there was no disparity signal. During a run of trials, the timing of binocular disparity onsets was manipulated (±300 ms) relative to colour changes according to the method of constant stimuli. Observers were required to indicate, by pressing one of two response buttons, if the disparity and colour changes appeared to be synchronous. The colour changes, from red (CIE 1931; *X*=0.63, *Y*=0.10, luminance=14.45) to blue (CIE 1931; *X*=0.15, *Y*=0.07, luminance=14.45), occurred near the mid-point of each 1.8 s stimulus presentation. The precise timing of the colour changes was randomized (±0.25 s) to ensure that the task did not become an interval bisection procedure. During each run of 390 trials, only one magnitude of horizontal disparity was sampled. The different trial runs were completed in a pseudorandom order by each observer in order to avoid practice effects.

Data for each observer provided distributions of perceived synchrony between colour changes and disparity onsets. Raised Gaussian functions were fitted to the distributions obtained for each observer for each of the four magnitudes of binocular disparity sampled. The peaks of the fitted functions were taken as perceived synchrony (PS) estimates. Perceived synchrony differences (PSDs) were calculated relative to the observers' PS estimate concerning the smallest disparity signal. Negative values indicate that disparity changes seemed to occur *sooner* relative to 0.07° disparity changes.

## 3. Results

### (a) Experiment 1: minimal exposures for signal detection vary with disparity magnitude

The minimal stimulus exposure necessary to detect a stimulus change is often related to signal strength. To ensure that this was true for our observers in this context, we first determined minimal exposure durations for detecting transient binocular disparity signals of differing magnitude. As can be seen in figure 1*b*, although there was some individual variation, the minimal exposure time required for an observer to detect a transient disparity signal was negatively related to disparity magnitude (*F*_3,12_=13.84, *p*<0.05)—a finding consistent with previous research (Uttal *et al*. 1975; Tyler 1991).

### (b) Experiment 2: rapid stimulus detections prompt faster reaction times

Larger disparity signals can be detected following shorter stimulus presentations than can smaller changes, suggesting that the former might be perceived more rapidly than the latter. We first tested this possibility by determining simple RTs for disparity changes of differing magnitude. As can be seen in figure 1*c*,*d*, RTs following the onset of a binocular disparity signal were negatively related to disparity magnitude (*F*_3,12_=3.97, *p*<0.05). This is consistent with the premise that information concerning large disparity changes becomes available for motor planning more rapidly than does information concerning smaller changes.

### (c) Experiment 3: large disparity changes seem to happen earlier than do smaller changes

Large disparity signals can be detected following shorter stimulus presentations than can smaller changes. They also prompt more rapid responses suggesting that perceptual information concerning large disparity changes is available more rapidly than that concerning smaller changes. However, the mechanisms that determine perceived timing might compensate for the variable temporal dynamics of different cortical analyses (Libet *et al*. 1979; Eagleman & Sejnowski 2000; Rao *et al*. 2001). We therefore determined subjective timing estimates for different magnitudes of disparity change. Disparity signal onsets were measured relative to colour changes. As can be seen in figure 1*e*,*f*, we found that the onsets of large horizontal disparity signals seemed to coincide with *earlier* colour changes relative to smaller disparity onsets (*F*_3,12_=14.76, *p*<0.05). As binocular disparity analyses originate in cortex (Barlow *et al*. 1967; Poggio & Fischer 1977), these findings clearly show that cortical analyses can influence perceived timing.

## 4. Discussion

We have shown that large binocular disparity signals can be detected more rapidly (experiment 1), be responded to sooner (experiment 2) and can seem to occur at earlier epochs (experiment 3) relative to smaller disparity signals. As analyses of binocular disparity originate in cortex, our findings clearly show that cortical processing can influence perceived timing.

As we mentioned in §1, it is already generally accepted that timing illusions can be instigated by differential delays between the physical exposure to a stimulus and the onset of activity in cortex (Roufs 1963; Wilson & Anstis 1969). However, the possibility that the variable dynamics of cortical processing might further contribute to the generation of timing illusions has remained a point of contention (Libet *et al*. 1979; Eagleman & Sejnowski 2000; Johnston & Nishida 2001; Rao *et al*. 2001; Nishida & Johnston 2002). The significance of our data is that we have used a stimulus attribute that can only be detected owing to an analysis that originates in cortex. Therefore, there is no ambiguity concerning whether or not our perceived timing effects can be attributed to the influence of cortical activity. This is not true for other apparent timing illusions (Mackay 1958; Roufs 1963; Wilson & Anstis 1969; Moutoussis & Zeki 1997) which involve stimulus characteristics processed extensively in subcortical structures, such as luminance, colour and motion.

In the interests of clarity, we will expand briefly on this last point. In primates, motion direction sensitivity is dependent upon cortical analyses (Dubner & Zeki 1971; Zeki 1974; Zeki *et al*. 1991). However, direction changes within a persistently moving stimulus (or at the onset of motion within a previously static surface) can induce transient signals in subcortical neurons that are sensitive to different rates of temporal modulation (Derrington & Lennie 1984; Lee *et al*. 1989; Merigan *et al*. 1991). Thus, it is conceivable that a timing illusion involving motion might be driven, at least in part, by subcortical analyses rather than cortical.

We place greatest emphasis on our direct measure of perceived timings (experiment 3) and on our measure of the minimal stimulus exposures necessary to detect transient disparity changes (experiment 1). Both are marked by qualitatively similar trends. Progressively shorter stimulus exposures are required to detect larger transient disparity changes (experiment 1). Similarly, larger disparity changes seem to occur at progressively earlier epochs (experiment 3).

There are, however, marked quantitative differences between these two measures. Experiment 1 involved an objective measure of sensitivity and was marked by relatively small effect sizes (largest effect approx. 29±13 ms). Experiment 3 used a subjective measure of perceived timing and was marked by larger effects (largest of approx. 58±17 ms). This difference is consistent with a very large literature showing that, in general, the strength of a sensory signal must exceed that of an objective threshold by some margin before the observers feel confident that they have detected that signal (Green & Swets 1966; Campion *et al*. 1983; Azzopardi & Cowey 1998). The difference between our measures may therefore indicate that subjective timing reflects the times at which observers become confident that they have detected a stimulus change (also see Arnold & Clifford 2002; Bedell *et al*. 2003; Ogmen *et al*. 2004). Presumably, such confidence arises following analyses subsequent to those that are minimally necessary for objective stimulus change detection.

We place less emphasis on our RT data (experiment 2). RTs might resemble a simple measure of sensory processing, but they are confounded with both the preparation and execution of motor responses. It can therefore be difficult to attribute, with confidence, RT variance to sensory processing. A further complication is posed by the characteristics of visual processing in the human brain. Specifically, visual processing is multifaceted and distributed across many different brain structures (Zeki 1978), so it is possible that a sensory processing difference could influence manual RTs but have no impact on perceived timing. However, our RT measures (experiment 2) were qualitatively similar to our direct measures of perceived timing (experiment 3) in that both suggested that information concerning large disparity changes becomes available at earlier epochs than that concerning smaller changes.

The systematic variance in perceived timing as a function of disparity change magnitude verifies a tight relationship between cortical processing and perceptual timing. Such changes are known to be detected via the temporal integration of cortical activity (Barlow *et al*. 1967; Uttal *et al*. 1975; Poggio & Fischer 1977; Tyler 1991). Far from compensating for this necessity, such that the changes seem to occur at the instigation of the period of integration (Libet *et al*. 1979; Eagleman & Sejnowski 2000; Rao *et al*. 2001; Moradi & Shimojo 2004; Amano *et al*. 2007), it seems that observers perceive the timing of such changes as having occurred at the point at which they became confident of change detection. The influence of the temporal integration of cortical activity is therefore even greater in this context than might be expected on the basis of objective sensory thresholds.

Cortical processing differences have previously been cited as an explanation for a variety of timing illusions (Moutoussis & Zeki 1997; Bartels & Zeki 1998; Whitney & Murakami 1998; Arnold *et al*. 2001; Bedell *et al*. 2003; Clifford *et al*. 2004; Arnold 2005). These proposals remain the focus of heated debate (Eagleman & Sejnowski 2000; Nishida & Johnston 2002; Moradi & Shimojo 2004; Amano *et al*. 2007). We will briefly discuss how our data relate to two of these persistent controversies.

When a flash is presented in physical alignment with a moving stimulus, the moving stimulus typically appears to be spatially advanced relative to the position of the flash—the well-known flash-lag illusion (Mackay 1958; Mateeff & Hohnsbein 1988; Nijhawan 1994). It seems that flash-lag magnitude can be modulated by inducing changes in neural processing speeds (Purushothaman *et al*. 1998; Patel *et al*. 2000; Ogmen *et al*. 2004). However, we believe that such differences usually play only a minor role in the generation of this illusion (see Arnold *et al*. 2003). Further, we do not believe that the flash lag provides a precise tool for measuring perceived timing. Flash-lag experiments usually derive a timing estimate from the apparent spatial relationship between a moving target and flash. The assumption underlying such analyses is that the illusion is driven primarily by a temporal offset rather than by a spatial bias. This assumption may be flawed (Eagleman & Sejnowski 2002, 2007). Thus, even when a flash-lag measure involves binocular disparity changes similar to those used here (Harris *et al*. 2006), we could not tell if any similarities or differences between the two datasets are necessarily systematic or coincidental.

Another debate surrounds the interpretation of a timing illusion concerning changes in colour and direction—repetitive colour changes can seem to precede physically synchronous direction changes (Moutoussis & Zeki 1997). In a conceptually similar manipulation to that used in experiment 3, it has been shown that the magnitude of this illusion varies as a function of the angular difference between the contrasted directions of motion (Arnold & Clifford 2002; Bedell *et al*. 2003; Amano *et al*. 2007). Originally these data were interpreted as showing that the temporal integration, and therefore the apparent timing, of direction changes are delayed in proportion to the degree of inhibition between direction selective V5 neurons (Arnold & Clifford 2002; Bedell *et al*. 2003). Readers should note that this interpretation presumes a tight relationship between the temporal integration of V5 cortical activity and the perceived timing of direction changes.

A similar dataset has since been interpreted quite differently (Amano *et al*. 2007). Specifically, it has been suggested that while there might be some relationship between the temporal integration of V5 activity and perceived timing, any such relationship is minimized by using the initial neural transient following a direction change as a temporal marker for the determination of perceived timing (Amano *et al*. 2007). This proposal is similar to others in which perceived timing is supposed to be more closely related to when cortical analyses commence rather to when they finish (Libet *et al*. 1979; Eagleman & Sejnowski 2000; Rao *et al*. 2001; Moradi & Shimojo 2004; Amano *et al*. 2007). We believe that the current data undermine such proposals.

Here, we have shown that the magnitude of a subjective timing illusion can be greater than that one would predict on the basis of the minimal exposure necessary for stimulus change detection. As the changes in question can only be detected via the temporal integration of cortical activity (Barlow *et al*. 1967; Uttal *et al*. 1975; Poggio & Fischer 1977; Tyler 1991), our data clearly show that considerable cortical processing can precede the neural event/s that determine subjective timing. Our data are therefore inconsistent with the notion that perceived timing is referred back (Libet *et al*. 1979; Moradi & Shimojo 2004; Amano *et al*. 2007) or is postdicted (Eagleman & Sejnowski 2000; Rao *et al*. 2001) to the time of the initial cortical response following a stimulus change.

Our data are more consistent with an alternate suggestion that perceived timing is a product of sensory analyses subsequent to those that are minimally necessary for sensory detection (Bartels & Zeki 1998; Zeki & Bartels 1998). The implication of this is that the determination of subjective timing might involve a further analysis of sensory events that have already begun to be perceived.

## Figures and Tables

**Figure 1:**
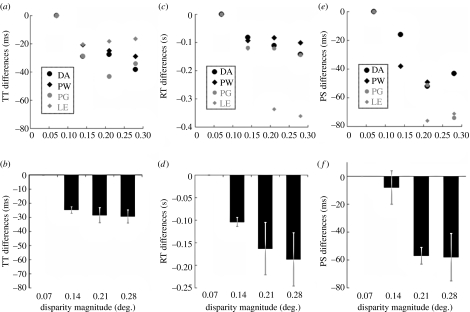
Scatter plots (*a*,*c*,*e*) show data for four individual observers. Bar graphs (*b*,*d*,*f*) show data averaged across these observers. Error bars show ±1 s.e. (*a*) Scatter plot showing temporal detection threshold differences (TT differences), relative to temporal threshold estimates concerning disparity changes of 0.07°, as a function of disparity change magnitude. (*b*) Average TT differences. (*c*) Scatter plot showing RT differences, relative to RTs for disparity changes of 0.07°, as a function of disparity change magnitude. (*d*) Average RT differences. (*e*) Scatter plot showing perceived synchrony (PS) differences, relative to PS estimates concerning disparity changes of 0.07°, as a function of disparity change magnitude. (*f*) Average PS differences.

## Supplementary Material

Supplementary file Tables showing the results of post-hoc tests contrasting measures of the dependent variables across different magnitudes of disparity change
