# Supplementary material for: Cortical processing and perceived timing
Source: Proc Biol Sci. 2007 Jul 10;274(1623):2331–6. doi: 10.1098/rspb.2007.0576 (PMC2211513; doi:10.1098/rspb.2007.0576)
Supplement: Supplementary file 1 [file rspb20070576s08.pdf]

a

Multiple Comparisons

Dependent Variable: Thres

Bonferroni

(I) Disp	(J) Disp	Mean Difference (I-J)	Std. Error	Sig.	95% Confidence Interval	
					Lower Bound	Upper Bound
.07	.14	24.68500*	5.27814	.003	8.0447	41.3253
	.21	28.37500*	5.27814	.001	11.7347	45.0153
	.28	29.36000*	5.27814	.001	12.7197	46.0003
.14	.07	-24.68500*	5.27814	.003	-41.3253	-8.0447
	.21	3.69000	5.27814	1.000	-12.9503	20.3303
	.28	4.67500	5.27814	1.000	-11.9653	21.3153
.21	.07	-28.37500*	5.27814	.001	-45.0153	-11.7347
	.14	-3.69000	5.27814	1.000	-20.3303	12.9503
	.28	.98500	5.27814	1.000	-15.6553	17.6253
.28	.07	-29.36000*	5.27814	.001	-46.0003	-12.7197
	.14	-4.67500	5.27814	1.000	-21.3153	11.9653
	.21	-.98500	5.27814	1.000	-17.6253	15.6553

*. The mean difference is significant at the .05 level.

b

Multiple Comparisons

Dependent Variable: RT_Diff

Bonferroni

(I) Disp	(J) Disp	Mean Difference (I-J)	Std. Error	Sig.	95% Confidence Interval	
					Lower Bound	Upper Bound
.07	.14	.10400	.05911	.624	-.0823	.2903
	.21	.16300	.05911	.104	-.0233	.3493
	.28	.18700*	.05911	.049	.0007	.3733
.14	.07	-.10400	.05911	.624	-.2903	.0823
	.21	.05900	.05911	1.000	-.1273	.2453
	.28	.08300	.05911	1.000	-.1033	.2693
.21	.07	-.16300	.05911	.104	-.3493	.0233
	.14	-.05900	.05911	1.000	-.2453	.1273
	.28	.02400	.05911	1.000	-.1623	.2103
.28	.07	-.18700*	.05911	.049	-.3733	-.0007
	.14	-.08300	.05911	1.000	-.2693	.1033
	.21	-.02400	.05911	1.000	-.2103	.1623

*. The mean difference is significant at the .05 level.

c

Multiple Comparisons

Dependent Variable: PS_Diff

Bonferroni

(I) Disp	(J) Disp	Mean Difference (I-J)	Std. Error	Sig.	95% Confidence Interval	
					Lower Bound	Upper Bound
.07	.14	8.50000	11.36469	1.000	-27.3293	44.3293
	.21	57.00000*	11.36469	.002	21.1707	92.8293
	.28	57.75000*	11.36469	.002	21.9207	93.5793
.14	.07	-8.50000	11.36469	1.000	-44.3293	27.3293
	.21	48.50000*	11.36469	.007	12.6707	84.3293
	.28	49.25000*	11.36469	.006	13.4207	85.0793
.21	.07	-57.00000*	11.36469	.002	-92.8293	-21.1707
	.14	-48.50000*	11.36469	.007	-84.3293	-12.6707
	.28	.75000	11.36469	1.000	-35.0793	36.5793
.28	.07	-57.75000*	11.36469	.002	-93.5793	-21.9207
	.14	-49.25000*	11.36469	.006	-85.0793	-13.4207
	.21	-.75000	11.36469	1.000	-36.5793	35.0793

*. The mean difference is significant at the .05 level.

SUPPLEMENTARY FILE LEGEND

SUPPLEMENTARY FILE 1: (a-c) Tables showing the results of post-hoc tests contrasting measures of the dependent variables across different magnitudes of disparity change. Tables are shown for contrasts of (a) Temporal Threshold Differences (TTDs) from Experiment 1, (b) Reaction Time Differences (RTs) from Experiment 2, and (c) for Perceived Synchrony Differences (PSDs) from Experiment 3.
